# Emergence of Human G2P[4] Rotaviruses in the Post-vaccination Era in South Korea: Footprints of Multiple Interspecies Re-assortment Events

**DOI:** 10.1038/s41598-018-24511-y

**Published:** 2018-04-16

**Authors:** Hien Dang Thanh, Van Trung Tran, Inseok Lim, Wonyong Kim

**Affiliations:** 1 Department of Microbiology, Chung-Ang University College of Medicine, Seoul, 06974 South Korea; 2 Department of Pediatrics, Chung-Ang University College of Medicine, Seoul, 06974 South Korea

## Abstract

After the introduction of two global rotavirus vaccines, RotaTeq in 2007 and Rotarix in 2008 in South Korea, G1[P8] rotavirus was the major rotavirus genotype in the country until 2012. However, in this study, an emergence of G2P[4] as the dominant genotype during the 2013 to 2015 season has been reported. Genetic analysis revealed that these viruses had typical DS-1-like genotype constellation and showed evidence of re-assortment in one or more genome segments, including the incorporation of NSP4 genes from strains B-47/2008 from a cow and R4/Haryana/2007 from a buffalo in India, and the VP1 and VP3 genes from strain GO34/1999 from a goat in Bangladesh. Compared to the G2 RotaTeq vaccine strain, 17–24 amino acid changes, specifically A87T, D96N, S213D, and S242N substitutions in G2 epitopes, were observed. These results suggest that multiple interspecies re-assortment events might have contributed to the emergence of G2P[4] rotaviruses in the post-vaccination era in South Korea.

## Introduction

Group A rotavirus (RVA) is the etiological agent primarily responsible for gastroenteritis in young humans and many other animal species. RVA, a member of the *Reoviridae* family, is an infectious virion that consists of a triple-layered icosahedral capsid containing a genome of 11 segments of double-stranded RNA in it. These segments encode six structural proteins (VP1–VP4, VP6, and VP7) and six non-structural proteins (NSP1–NSP6)^1^. Recently, a genotyping system, based on the nucleotide sequences of all 11 gene segments, was established, and a rotavirus classification working group (RCWG) was formed^2^. This classification system has been used to describe interspecies transmission and re-assortment of rotavirus strains from humans and other animals^3^. In this classification system, a cut-off nucleotide percentage in each of the rotavirus gene segments is used to distinguish between the different genotypes^4,5^. According to this system, 27 G (glycoprotein, VP7), 37 P (protease sensitive, VP4), 18 I (intermediate capsid shell, VP6), 9 R (RNA polymerase, VP1), 9 C (core shell, VP2), 8 M (methyltransferase, VP3), 19 A (interferon antagonist, NSP1), 10 N (NTPase, NSP2), 12 T (translation enhancer, NSP3), 15 E (enterotoxin, NSP4), and 11 H (phosphoprotein, NSP5) genotypes have been established^6–8^.

Full genome analyses of human RVA strains have revealed that most human rotaviruses are classifiable into at least two major genogroups, i.e., the Wa genogroup with a Wa-like backbone G1 (or 3, 4, 9)-P[8]-I1-R1-C1-M1-A1-N1-T1-E1-H1 genotype constellation, or the DS-1 genogroup with a DS-1-like backbone G2-P[4]-I2-R2-C2-M2-A2-N2-T2-E2-H2 genotype constellation. Each year, multiple variants co-circulate in a given area, hence changing the strain prevalence^9,10^. In recent years, a world-wide increase in the circulation of G2P[4] strains has been reported^11–15^. Moreover, the recently detected G2 RVAs that carry hallmark nonsynonymous changes within the antigenic domain of VP7 have become prevalent^16^. The accumulation of these VP7 mutations may have improved the fitness of G2 viruses by allowing neutralisation escape, thereby explaining the increased incidence of G2P[4]-associated disease^16^.

In 2006, two RVA vaccines were licensed in many countries around the world. In South Korea, the pentavalent human-bovine re-assortant vaccine (RotaTeq, Merck & Co. Inc., West Point, PA) and the live-attenuated monovalent human G1P[8] vaccine (Rotarix, GlaxoSmithKline Biologicals, Rixensart, Belgium) were introduced in September 2007 and July 2008, respectively^17^. RotaTeq contains five human rotavirus genotypes (G1–G4, P[8]) and bovine rotavirus genotypes G6 and P[5] with a bovine WC3 backbone^18^. Since none of the currently licensed RVA vaccines contain the P[4] genotype, it is important to monitor the prevalence of the G2P[4] genotype in the human population and understand the genetic constellations of P[4] RVA strains and their relationship with the more prevalent P[8] or P[6] genotyped RVA strains.

A previous study had shown the evolution of G2P[4] strains as a series of stepwise changes in lineages at the whole genome level^19^. Because of the current dominance of G2P[4] rotaviruses in Korea, a large-scale whole-genome study of these rotavirus strains would provide significant information about the evolution of RVAs, which is highly relevant to vaccine design and implementation; however, to our knowledge, there is no G2P[4] strain in Korea, whose whole genome has been sequenced. Therefore, the complete genomes of twelve G2P[4] rotavirus strains were sequenced in this study. These strains were isolated from stool specimens collected from children with gastroenteritis in Seoul, Korea, between 2010 and 2015. The data obtained in the study were compared with that of rotavirus strains from other parts of the world.

## Results

### Prevalence of common genotypes among the G2P[4] strains in South Korea

**Figure 1:**
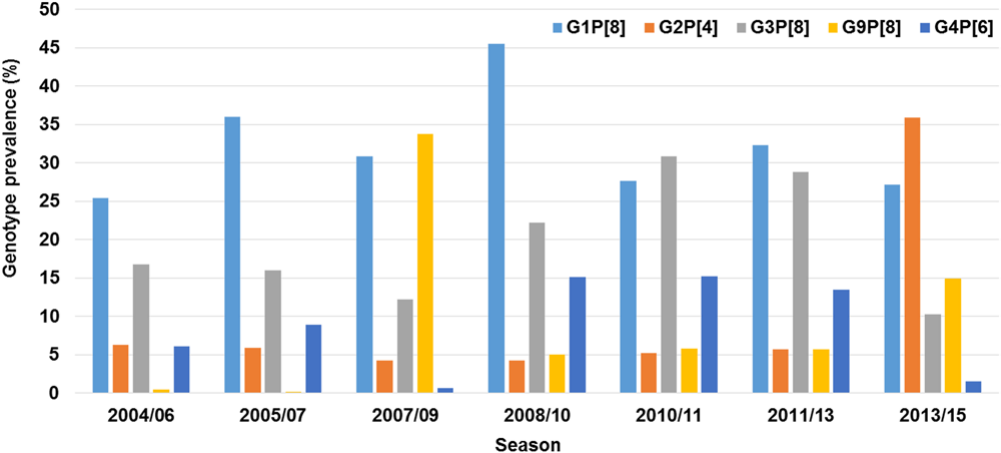
Annual rates of rotavirus and G2P4 occurrence in Seoul, South Korea, from 2004 to 2015.

Of the 1,126 diarrhoeal specimens, collected from children under five years of age, presenting acute gastroenteritis at Chung-Ang University Hospital between 2013 and 2015, 195 (17.3%) were found positive for rotaviruses. The annual RVA detection rates were 16.2%, 20.9%, and 14.9% in 2013, 2014, and 2015, respectively. All 195 rotavirus-positive samples were subjected to both G and P genotyping. The most common G and P combinations were G2P[4] (n = 70, 35.9%), G1P[8] (n = 53, 27.2%), G9P[8] (n = 29, 14.9%), and G3P[8] (n = 20, 10.3%). Interestingly, the number of G2P[4]-infected children during 2013–2015, with a peak in 2013, was notable, as it represented a greater number of G2P[4] infections compared to that observed in previous studies between 2004 and 2013 (Fig. 1). Five other unusual rotavirus strains, G4P[6] (n = 3, 1.55%), G2P[6] (n = 2, 1%), G3P[4] (n = 2, 1%), G4P[4] (n = 2, 1%), and G9P[4] (n = 1, 0.5%), were identified. Rare G3P[9] (n = 2, 1%) and G11P[25] (n = 1, 0.5%) RVAs were also observed. In addition, 3.65% and 1.5% of the RVAs detected could not be G and P genotyped, respectively.

### Genotype constellation and phylogenetic analyses of the G2P[4] RVA strains

A multiple sequence alignment and phylogenetic analysis of the VP7 genes in the 91 strains detected between 2010 and 2015 (after the introduction of the rotavirus vaccines) showed the Korean G2 strains clustered into three groups: the G2 lineage V (2, 2.2%) and sub-lineages IVa-1 (80, 89.9%) and IVa-3 (7, 7.9%) of G2 lineage IV (Fig. S1). Twelve Korean G2P[4] strains, detected in six different years of G2P[4] circulation, 2010 (CAU10-01 and CAU10-02), 2011 (CAU11-03 and CAU11-04), 2012 (CAU12-05 and CAU12-06), 2013 (CAU13-07 and CAU13-08), 2014 (CAU14-09 and CAU14-10), and 2015 (CAU15-11 and CAU15-12), were selected randomly from the three groups as representatives of the various VP7 lineages. Based on nucleotide sequence identities, all 12 Korean strains possessed the archetypal DS-1-like genome constellation G2-P[4]-I2-R2-C2-M2-A2-N2-T2-E2-H2. The 11 genome segments of the Korean G2P[4] strains showed low nucleotide sequence similarities, ranging from 87.46% for VP3 to 90.03% for NSP4.

**Figure 2:**
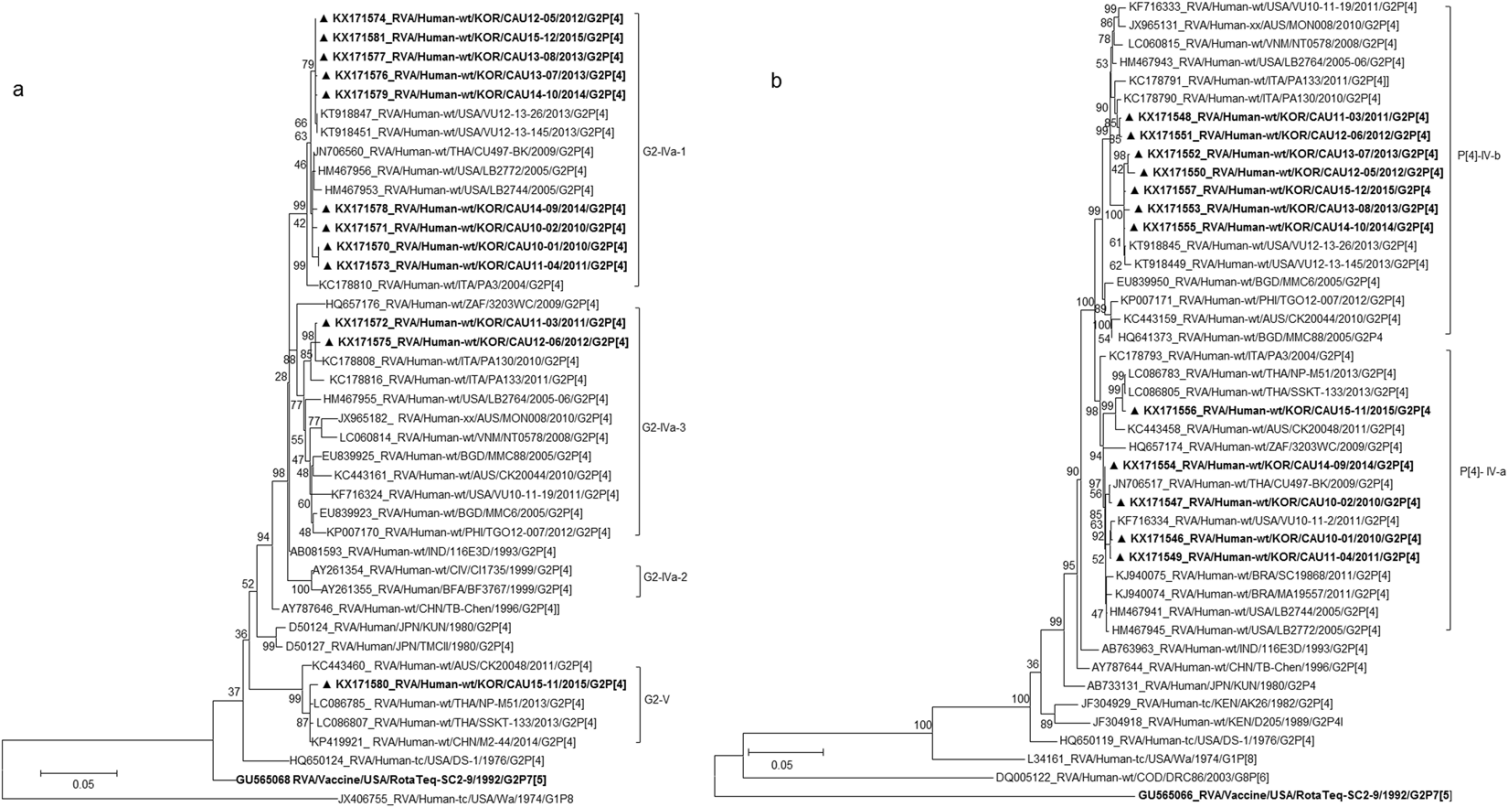
Phylogenetic tree constructed from the nucleotide sequences of VP7 (**a**) and VP4 (**b**) genes in Korean and representative RVA G2P[4] strains. Sequences obtained in the present study are indicated by a black triangle.

The VP7 gene tree (Fig. 2a) showed that, with the exception of CAU15-11, all Korean strains were classified into lineage G2-IV, in either of the two sub-lineages, G2-IVa-1 and G2-IVa-3, exhibiting maximum nucleotide sequence identities (98.9–99.7%) with co-circulating human G2P[4] strains VU12-13-26, CU497-BK, and PA130. The CAU15-11 strain in lineage G2-V clustered with human strains SSKT-133 (Thailand) and M2-44 (China). In lineage G2-IV, G2P[4] strains were clustered with strains CU497-BK and VU12-13-26 in G2 sub-lineage IVa-1. G2P[4] strains in sub-lineage IVa-3 were shown to be closely related to human G2P[4] strain PA130 (Italy). Among Korean G2 strains, VP7 nucleotide and amino acid sequence identities were 92.8–100.0% and 96.0–100.0%, respectively.

The VP4 genes showed 96.2–97.5% sequence identities with the cognate genes of Malawian human strain TB-Chen (G2P[4]). In phylogenetic analysis, the VP4 gene clustered into two sub-lineages of IV (IV-a and -b) (Fig. 2b). Sub-lineage IV-b included seven of the twelve G2P[4] strains analysed, together with G2P[4] strains previously detected in the USA and Italy. Five strains in sub-lineage IV-a were closely related to strains SSKT-133, CU497-BK, and VU10-11-2, which were detected in Thailand and the USA during 2009–2013. Among the Korean P[4] strains, VP4 nucleotide identities were 96.5–99.8%.

**Figure 3:**
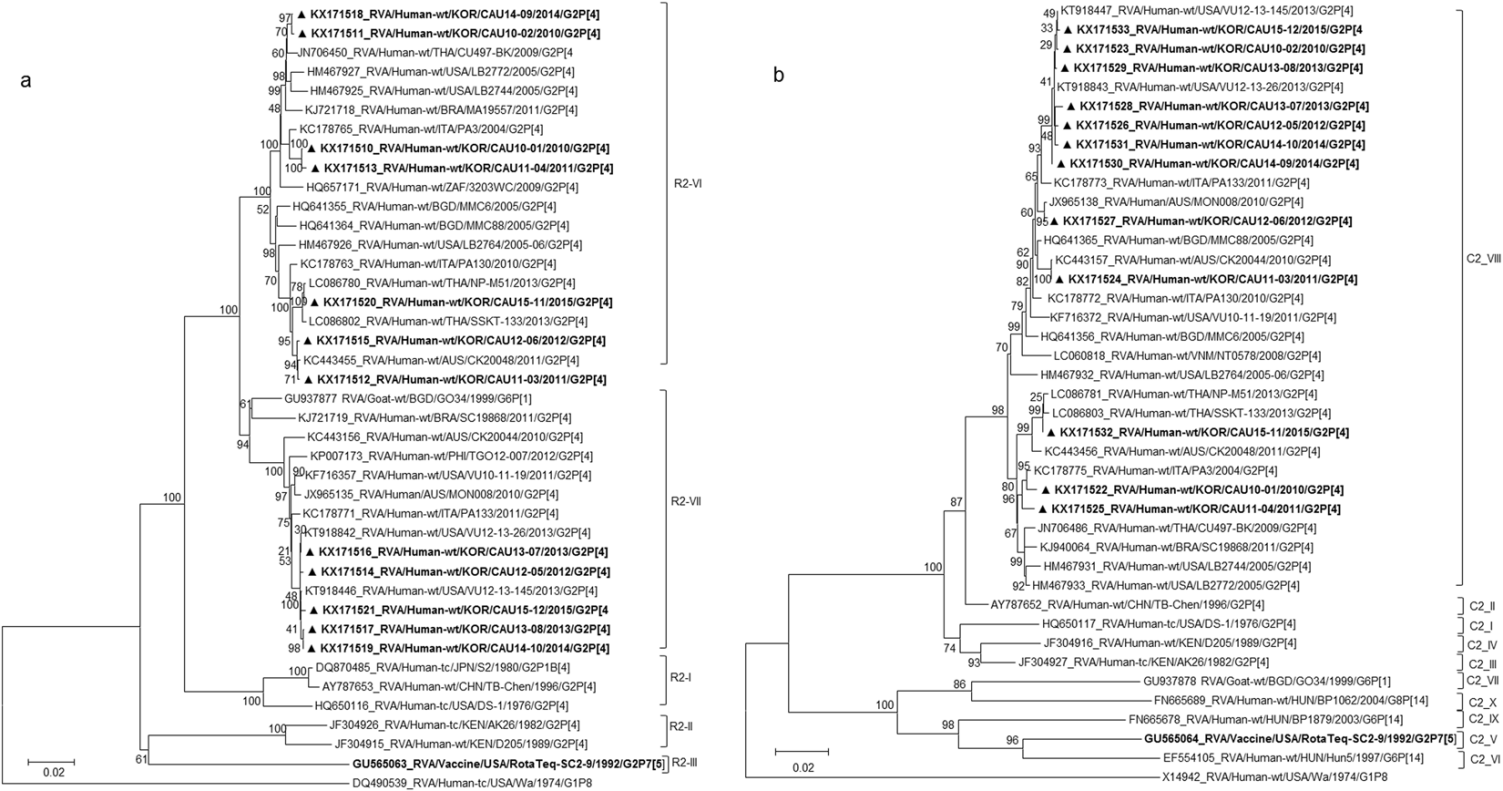
Phylogenetic tree constructed from the nucleotide sequences of VP1 (**a**) and VP2 (**b**) genes in Korean and representative RVA G2P[4] strains. Sequences obtained in the present study are indicated by a black triangle.

The VP1 genes in the 12 G2 strains belonged to the R2 genotype, with sequence identities ranging from 94.3% to 99.9%, and differentiated into two clusters (VI and VII) (Fig. 3a). Phylogenetic analysis also indicated that strains in sub-lineages R2-VI clustered with human strains frequently detected in other countries (Fig. 3a). In sub-lineage R2-VII, four Korean strains were shown to cluster with USA strains VU12-13-26 and VU12-13-145. A strain isolated from a goat in Bangladesh in 1999 (RVA/Goat-tc/BGD/GO34/1999/G6P[1]) belonged to the same sub-lineage (Fig. 3a).

The VP2 genes were highly conserved and showed high nucleotide sequence identities (97.2–99.9%) with the cognate genes of human strains (VU12-13-26, VU12-13-45, SSKT-133, and PA3) from the USA, Thailand, and Italy (Fig. 3b). Further, the VP2 genes were located in a cluster having several common human strains in sub-lineage C2-VIII (Fig. 3b).

**Figure 4:**
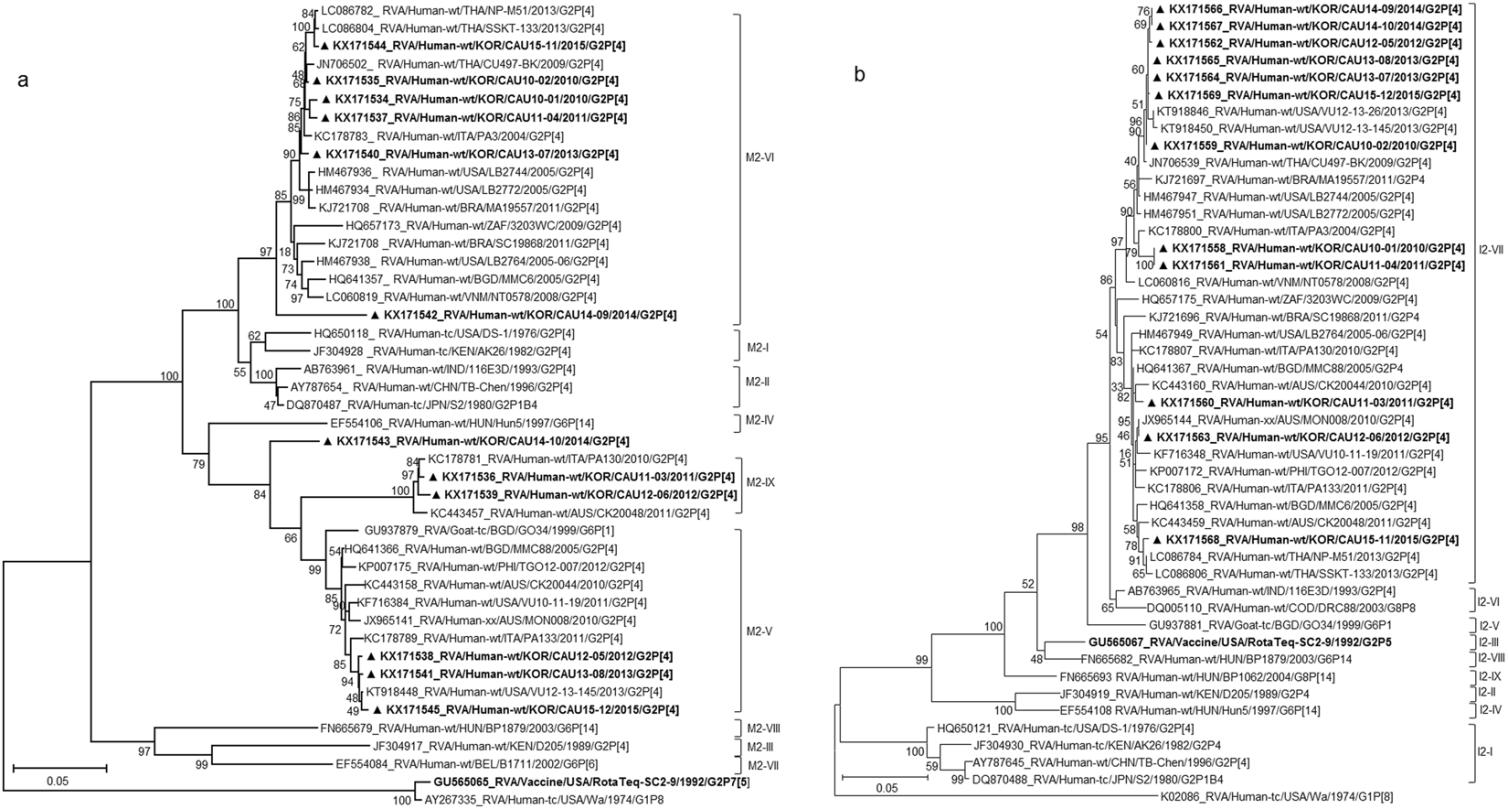
Phylogenetic tree constructed from the nucleotide sequences of VP3 (**a**) and VP6 (**b**) genes in Korean and representative RVA G2P[4] strains. Sequences obtained in the present study are indicated by a black triangle.

Phylogenetic analysis of the VP3 gene showed that all Korean strains clustered into lineage M2, sub-lineages V, VI, and IX, along with contemporary G2 strains from other countries, and showed nucleotide identities between 87.5–99.4% with the other strains (Fig. 4a). Although three strains (CAU15-12, CAU13-08, and CAU12-05) clustered with other human strains, VP3 in these strains was found to share a common ancestor with RVA/Goat-tc/BGD/GO34/1999/G6P[1], a strain isolated from a goat in Bangladesh in 1999 (Fig. 4a). Korean strain CAU14-10 was classified into lineage M2 and shown to be closely related to strain Hun5/1997/G6P[14] in the M2-IV sub-lineage, PA130/2010/G2P[4] in the M2-IX sub-lineage, and MMC88/2005/G2P[4] in the M2-V sub-lineage, with nucleotide identities of 89.2%, 90.6%, and 95.4%, respectively).

In the VP6 gene tree, all strains were highly conserved and showed high nucleotide identities (96.9–99.9%) with strains frequently detected in other countries. Phylogenetic analysis also indicated that G2P[4] clustered with strains in the I2 genotype of human-origin rotaviruses, including VU12-13-26, PA3, CK20044, MON008, and SSKT-133 (Fig. 4b).

**Figure 5:**
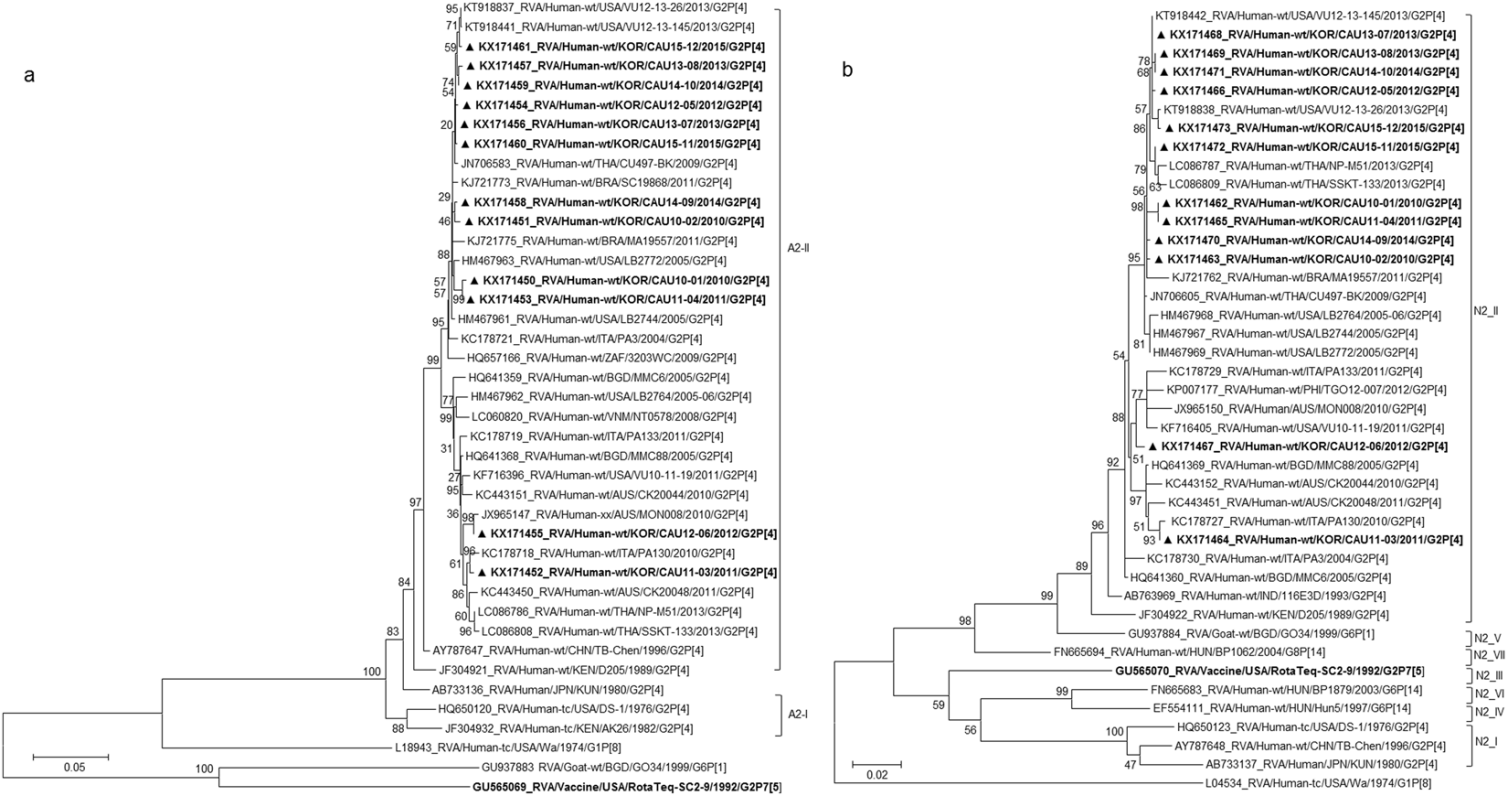
Phylogenetic tree constructed from the nucleotide sequences of NSP1 (**a**) and NSP2 (**b**) genes in Korean and representative RVA G2P[4] strains. Sequences obtained in the present study are indicated by a black triangle.

**Figure 6:**
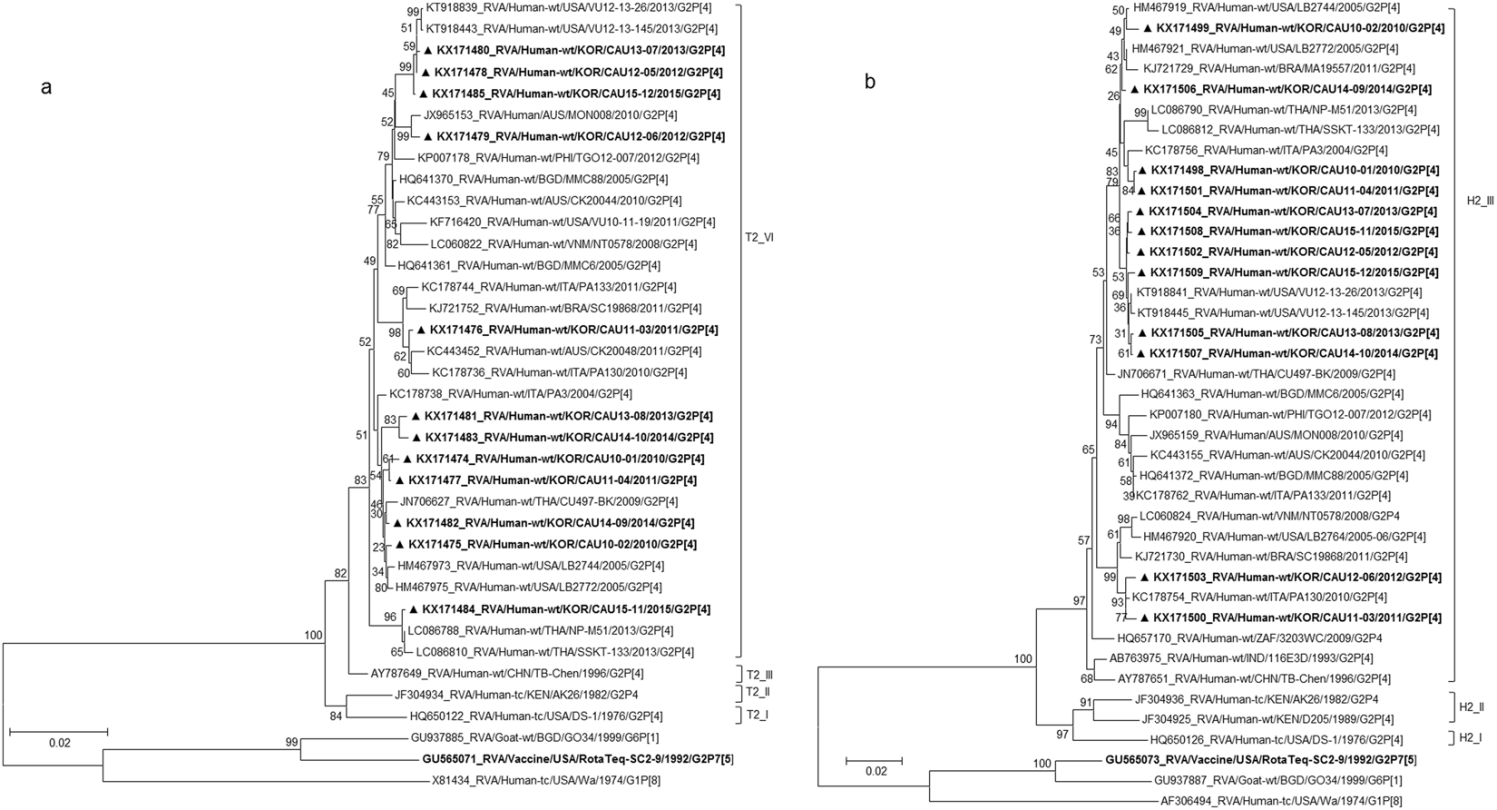
Phylogenetic tree constructed from the nucleotide sequences of NSP3 (**a**) and NSP5 (**b**) genes in Korean and representative RVA G2P[4] strains. Sequences obtained in the present study are indicated by a black triangle.

The NSP1, NSP2, NSP3, and NSP5 genes (A2, N2, T2, and H2, respectively) were highly conserved, with nucleotide identities of 96.9–99.8%, 97.5–100%, 96.7–99.9%, and 97.3–99.9%, respectively, and were located in a single cluster in the respective phylogenetic trees (A2-II, N2-II, T2-VI, and H2-III) (Figs 5a,b and 6a,b). In addition, the Korean G2P[4] strains were found to be closely related to human G2P[4] strains detected in the USA (2005, 2013), Italy (2010, 2011), Australia (2010), Brazil (2011), and Thailand (2009, 2013) (Figs 5a,b and 6a,b).

**Figure 7:**
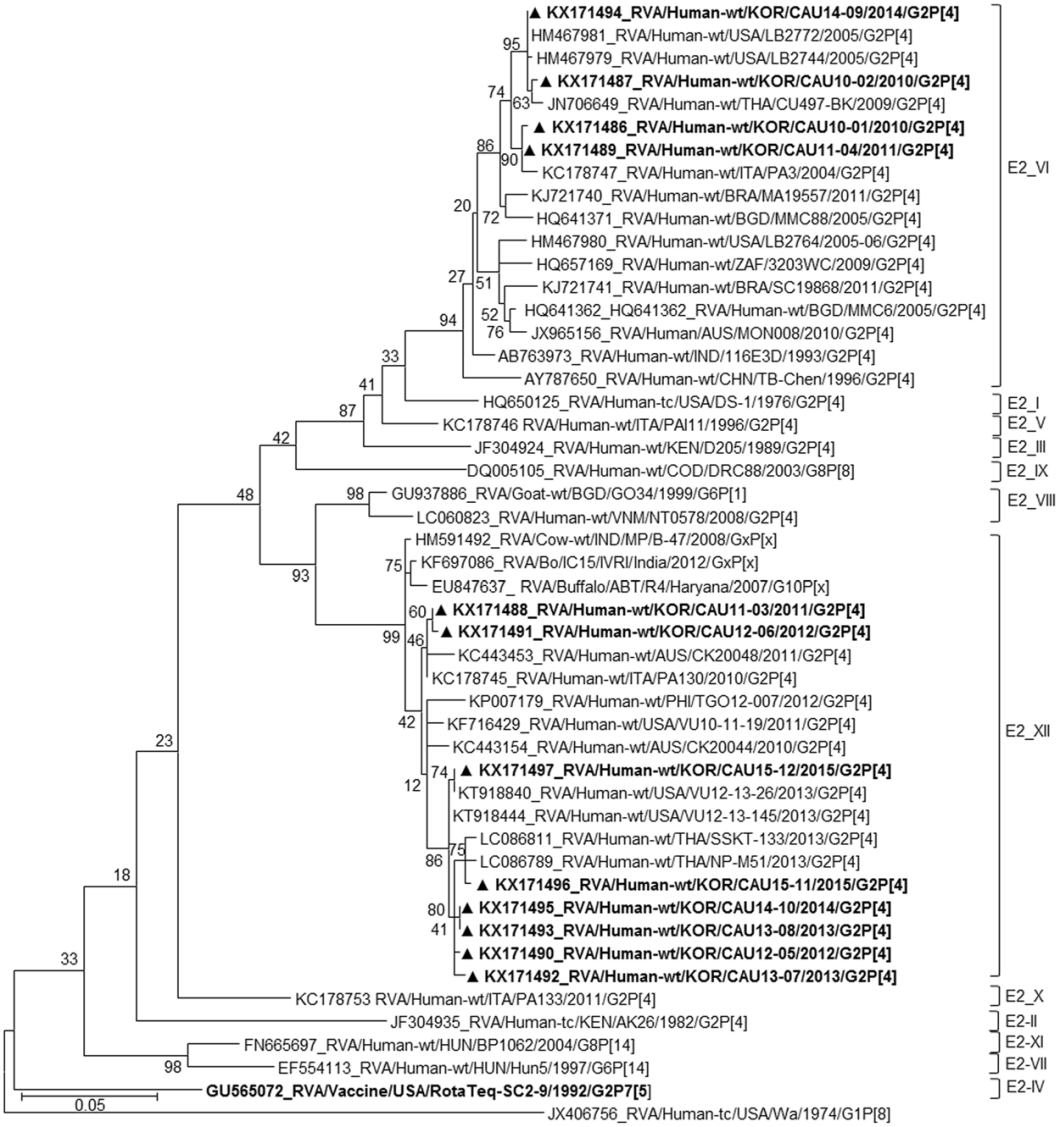
Phylogenetic tree constructed from the nucleotide sequences of NSP4 genes in Korean and representative RVA G2P[4] strains. Sequences obtained in the present study are indicated by a black triangle.

Based on the NSP4 gene, strains appeared on two branches with human rotavirus-like (sub-lineage E2-VI) and animal rotavirus-like strains (sub-lineage E2-XII). Genes of sub-lineage E2-VI clustered with strains LB2772, CU497-BK, and PA3, which were isolated in the USA (2005), Thailand (2009), and Italy (2004) (Fig. 7). Genes of sub-lineage E2-XII fell into a group heavily populated by rotavirus of animal origin, for example, RVA/Cow-wt/IND/MP/B-47/2008/GxP[x] and RVA/Buffalo/ABT/R4/Haryana/2007/G10P[x], which were obtained in 2008 and 2007, respectively, in India. Strains in sub-lineage E2-XII also shared a common ancestor with several other human strains, including SSKT-133, VU12-13-26, and CK20048.

In the phylogenetic analysis, strains CAU11-03, CAU12-05, CAU12-06, CAU13-07, CAU13-08, CAU14-10, CAU15-11, and CAU15-12 were shown to be re-assortants, with one or more gene segments (VP1, VP3, and/or NSP4) from RVAs, possibly of non-human (animal) origin. In the case of VP3 gene, strains CAU15-12, CAU13-08, and CAU12-05 grouped with VU12-13-145. All the VP3 genes shared a common ancestor with RVA/Goat-tc/BGD/GO34/1999/G6P[1]. The VP1 gene of strain RVA/Goat-tc/BGD/GO34/1999/G6P[1] also appeared to be closely related to Korean G2P[4] strains CAU12-05, CAU13-07, CAU13-08, CAU14-10, and CAU15-12. On the other hand, the NSP4 genes of CAU11-03, CAU12-05, CAU12-06, CAU13-07, CAU13-08, CAU14-10, CAU15-11, and CAU15-12 were closely related to strains RVA/Cow-wt/IND/MP/B-47/2008 and RVA/Buffalo/ABT/R4/Haryana/2007/G10 from India.

Furthermore, several intra-genogroup re-assortment events were identified in G2P[4] strains co-circulating during the 2010–2015 season. Strains CAU14-09 and CAU14-10 clustered in the same sub-lineage, based on the analysis of eight genes, VP2, VP4, VP6, VP7, NSP1–NSP3, and NSP5. The remaining three genes, VP1, VP3, and NSP4, of these strains clustered in different sub-lineage groups. Similarly, with the exception of NSP1, which was closely related to VU12-13-26 from the USA, the other 10 genome segments of CAU15-11 grouped closely with those of the Thai SSKT-133 strain (Fig. 5a). Overall, the great genomic diversity of DS-1-like G2P[4] strains seems to have been generated through re-assortment events between human and animal strains.

### Comparison of VP7 amino acid sequences with those of vaccine strains

**Figure 8:**
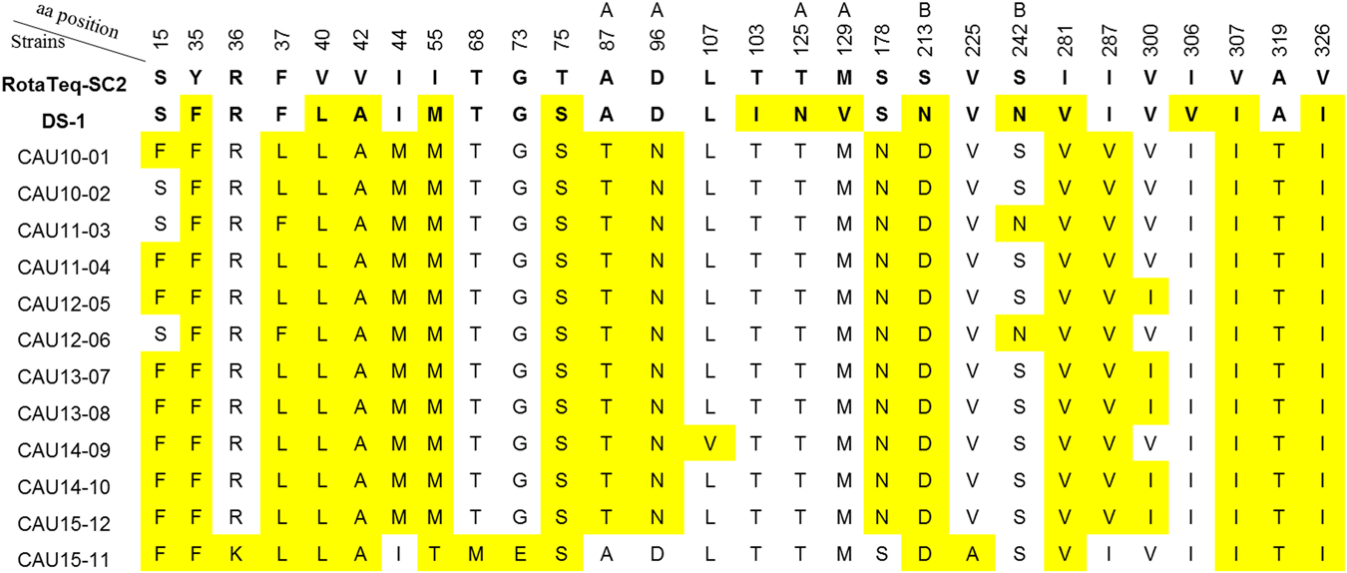
Alignment of the VP7 amino acid sequences in 12 Korean G2P[4] rotaviruses with those of the G2 RotaTeq vaccine and DS-1 strains. Positions of antigenic epitopes 7-1a, 7-1b, and 7-2 are indicated by A, B, and C, respectively. Amino acid numbering is based on the DS-1 strain sequence.

To identify substitutions in neutralisation epitopes and variable regions, the deduced amino acid sequences of the 12 G2P[4] strains, analysed in this study, were aligned with those of representative strains, including the G2 vaccine strain SC2-9 (lineage II) and prototype G2 strain DS-1 (lineage I; residue numbering based on the DS-1 sequence). The amino acid sequences of VP7 of the Korean G2P[4] strains exhibited 17–24 and 13–21 mismatches with the corresponding sequences in the G2 component of RotaTeq and the prototype G2P[4] strain DS-1, respectively. Among the amino acids in the VP7 epitopes, all Korean G2P[4] strains, (except CAU15-11) showed 3–4 substitutions (two in 7-1a and one or two in 7-1b) relative to that of the RotaTeq G2P[5] (Fig. 8). These differences were observed at positions 87 (AT; alanine to threonine) and 96 (DN; aspartic acid to asparagine) in the 7-1a epitope and at positions 213 (SD; alanine to threonine) and 242 (SN; serine to asparagine) in 7-1b epitope of strains CAU11-03 and CAU12-06, whereas the amino acid residues in the 7-2 epitope were all conserved. In contrast, three amino acid residues (87, 96, and 242) in the 7-1a and 7-1b epitopes were conserved in CAU15-11 and SC2-9. The analysis also showed that the Korean G2 strains contained three amino acid substitutions (V40L, V42A, and I44M) in one or two linear cytotoxic T lymphocyte epitopes at amino acid positions 40–52) when compared to the SC2-9 sequence.

## Discussion

The prevalence of rotavirus genotypes varies depending on the socio-economic status of the population under study, climates of different countries, and histo-blood group antigen types in different individuals and populations around the globe^20,21^. According to long-term surveillance of rotavirus genotypes in France, the United Kingdom, other western European countries^22^, and Thailand^23^, G1P[8] was the most prevalent rotavirus genotype during 2007–2014, whereas G3P[8] was dominant in China and Japan^11^. However, in Korea, a different trend of rotavirus genotypes has been observed. According to the current study, G2P[4] was the dominant genotype (35.9%) for three years in one region, followed by G1P[8], G9P[8], and G3P[8]. Although the reason for the prolonged dominance of G2P[4] in Korea remains uncertain, G2P[4] human rotaviruses should be considered a primary target for rotavirus vaccines in countries like South Korea.

Over the last 12 years, surveillance in Seoul, South Korea has shown that rotavirus frequencies vary across seasons. Before the introduction of a rotavirus vaccine, the frequency of genotypes varied over time, and G2P[4] RVAs were infrequently detected. G1P[8], G3P[8], and G4P[6] were the strains most frequently circulating in Seoul between 2004 and 2007^24,25^. In the initial years of rotavirus vaccination era (2007–2013), G9P[8] was predominant during 2007–2009, G1P[8] resurged as the predominant strain during 2008–2010 and 2011–2013, and G3P[8] was predominant during 2010–2011 (Fig. 1)^26–29^. Previous studies on different provinces in Korea revealed that G2P[4] was the fourth most common type, following G1P[8], G3P[8], and G9P[8], between 2006 and 2012, accounting for approximately 2.71% of circulating strains^11^. In contrast, the current study clearly indicates the emergence of G2P[4] strains, representing more than 35.9% of rotavirus infections in Seoul from March 2013 to August 2015. G2P[4] genotype was predominant in many other countries, including Belgium, Austria, Brazil, and Australia, where the Rotarix vaccine was introduced^11–15,30,31^. However, the samples in this study were collected from Seoul, where vaccination in children is optional (by parental choice) and administered according to opportunity and availability, with RotaTeq being more popular than Rotarix^32^. G2P[4] strains were found to be predominant even after the introduction of RotaTeq in Australia, Nicaragua, and other South and Central American countries, even though there was no established rotavirus vaccination program at this time^9,11,12,33,34^. These data suggest that the genetic changes observed in this study might have resulted from natural variation or re-assortment events between human and animal strains.

The twelve Korean G2P[4] RVA strains, analysed in the current study, had a complete DS-1-like background; however, other genetic variants were also observed to circulate in Korea. Several of the dendrograms, such as those for NSP1–5, VP1–4, and VP6, suggested re-assortment among the co-circulating strains. All genes, except for VP1, VP3, and NSP4, in the Korean G2P[4] strains, were closely related to those of other human RVA strains, but showed difference from the prototype DS-1 strain, with nucleotide identities between 86.2 and 96%. VP1, VP3, and NSP4 were the most divergent RVA genes, and appeared to share a distant common ancestor with strains obtained from a goat, buffalo, and cow, respectively. For example, strains CAU15-12, CAU13-08, and CAU12-05 (VP3), and CAU12-05, CAU13-07, CAU13-08, CAU14-10, and CAU15-12 (VP1) appear to share a distant common ancestor with strain RVA/Goat-tc/BGD/GO34/1999/G6P[1] from a goat (97% identity). Strains CAU11-03, CAU12-05, CAU12-06, CAU13-07, CAU13-08, CAU14-10, CAU15-11, and CAU15-12 (NSP4) showed a close phylogenetic relationship with RVA/Buffalo/ABT/R4/Haryana/2007/G10P[x] and RVA/Cow-wt/IND/MP/B-47/2008/GxPx (97.5–99.3% identity). Similar constellations, with one (or sometimes two) genes from other species, have recently been described for G2P[4] RVA strains detected in the USA, Italy, and Brazil^35–37^. Re-assortment between animal and human strains is predicted to rapidly alter RV diversity by introducing novel gene constellations^10,38,39^. However, available data regarding typical goat, buffalo, and cow RVA strains are limited, making it difficult to determine the species of origin for genome segments from non-human animal strains incorporated into human RVA strains.

The efficacy of vaccines against diarrhoea caused by various rotavirus genotypes has been well studied and ranges between 61% and 88% for Rotarix and 88% and 95% for RotaTeq, depending on the virus genotype^40^. In recent vaccine trials, both Rotarix and RotaTeq were shown to be equally effective against G1, G3, G4, and G9 strains with P[8] specificity^41^. The efficacy of the Rotarix vaccine against G2 strains is somewhat lower than that of RotaTeq, since the latter might contain a G2 antigen that is highly similar to the circulating G2 strains^42^. However, the Korean strains, analysed in this study, did not show a close phylogenetic relationship with the G2 VP7 gene in RotaTeq strain SC2–9. Furthermore, due to the number of amino acid changes and the absence of a P[4] component, the RotaTeq vaccine might also be less effective in inducing the production of antibodies capable of protecting against the circulating G2P[4] strains^42^.

The present study compared the amino acid motifs in the neutralising epitopes of VP7 proteins across the circulating Korean G2P[4] rotaviruses, their prototype DS-1 strains, and available vaccine strains. The VP7 protein contains three antigenic epitopes, 7-1a, 7-1b, and 7-2, which comprise of 29 amino acids from positions 87 to 291^43^. Several previous studies have indicated that substitutions at these positions, with or without changes in glycosylation, can change the antigenicity of human rotaviruses and allow them to escape host immunity^44–46^. Four notable changes were observed within the antigenic epitopes 7-1a and 7-1b: A87T, D96N, S213D, and S242N. These positions are commonly known as immunodominant sites of the VP7 protein, and are critical for reactions with neutralising monoclonal antibodies. Lazdins *et al*.^47^ reported that rotaviruses with a mutation in the antigenic epitope 7-1b showed a 10-fold increase in resistance to neutralisation by antiviral antiserum. Amino acid substitutions at these positions were found to be associated with an inability to serotype a strain and are responsible for antigenic changes^48^. Recently, amino acid substitutions in the three main antigenic regions, especially at positions 96 and 213 were postulated to be associated with the emergence of rotavirus G2 in Korea during 2010–2015. This study represents the first large-scale whole-genome study to understand the evolution of rotaviruses after the introduction of rotavirus vaccines into the Korean immunisation program. Continuous molecular epidemiological monitoring of RVAs will be necessary to prevent and control them and to determine the need for the inclusion of an RVA vaccine in the national immunisation program.

## Materials and Methods

### Ethics statement

The stool samples were collected as per protocol number #2010-10-02, approved by the Human Subjects Institutional Review Board (IRB) of Chung-Ang University College of Medicine, Seoul, Korea. All experiments were performed in accordance with IRB guidelines and regulations. For children enrolled in this study, written informed consent was obtained from parents or legal guardians. This consent included authorisation to use the data for future research purposes.

### Stool sample preparation

From March 2013 through August 2015, 1,126 stool samples were collected from children, under five years of age, presenting acute gastroenteritis at Chung-Ang University Hospital in Seoul, Korea. Acute gastroenteritis was defined in this study as increased stool frequency (i.e., at least three loose and watery stools within a 24-h period), with or without vomiting and fatigue, occurring within the preceding 48 h. Approximately 10% suspensions of stool samples were prepared by vortexing stool sample (0.1 g) with phosphate-buffered saline (1 mL) (PBS; pH 7.4). The stool sample suspensions were centrifuged at 12,000x *g* for 15 min, and the supernatants were used as the faecal suspensions.

### RNA extraction

Viral RNA was extracted from the faecal suspensions using a QIAamp Viral RNA Mini Kit (Qiagen, Hilden, Germany) according to the manufacturer’s instructions. Extracted viral RNA was stored at −70 °C until it was used in reverse transcription-polymerase chain reactions (RT-PCRs).

### Genotyping of rotaviruses

To genotype RVA strains, multiplex semi-nested PCR assays using two sets of primers, for VP7 and VP4, were performed. The VP7 gene was amplified from dsRNA genomes using primers Beg9 and End9 under conditions described previously^49^. G genotyping was performed using primer End9 and primers aBT1, aCT2, aET3, aDT4, aAT8, aFT9, G10, and G12, specific for G types 1, 2, 3, 4, 8, 9, 10, and 12, respectively^49–51^ (20–22). To amplify the VP4 gene, the consensus primers Con3 and Con2 were employed, and a semi-nested PCR was performed for P genotyping the virus using primer Con3 and primers 1T-1, 2T-1, 3T-1, 4T-1, and 5T-1, specific for P types P[8], P[4], P[6], P[9], and P[10], respectively^52^. The first PCR products were purified using a QIAquick PCR Purification Kit (Qiagen GmbH, Hilden, Germany). Nucleotide sequencing was conducted by Macrogen (Seoul, Korea) using a BigDye Terminator Cycle Sequencing Kit and an automated DNA sequencer (Model 3730; Applied Biosystems, Waltham, MA). Each amplicon was sequenced in both forward and reverse directions, and the sequences were assembled using BioEdit software (http://www.mbio.ncsu.edu/bioedit/bioedit.html). Genotype assignment for each gene was performed using BLAST (http://blast.ncbi.nlm.nih.gov/Blast.cgi) and RotaC 2.0 (http://rotac.regatools.be/).

### Whole genome sequencing

G2P[4] strains, representative of the surveillance period, were selected as described previously^26,27^, and the G2P[4]-positive samples having sufficient amount of the original faecal sample available were subjected to whole genome analysis. Nearly full-length nucleotide sequences, including the 5′ and 3′ termini of gene segments encoding VP7, VP4, VP6, VP1, VP2, VP3, NSP1, NSP2, NSP3, NSP4, and NSP5/6 of the selected RVA strains, were obtained as described elsewhere^53^. RT-PCRs were performed using a One Step RT-PCR Kit (Qiagen). Each PCR product was confirmed, purified, and sequenced as described above. The genotypes of the G2P[4] RVA strains were determined according to the recommendations of the RCWG, using the RotaC online classification tool (http://rotac.regatools.be)^54^. The nucleotide sequences were deposited in GenBank under accession numbers KX171450–KX171581.

### Phylogenetic analysis

The nucleotide sequences of the G2P[4] strains were compared with representative rotavirus sequences available in the GenBank database. Phylogenetic trees were constructed using the neighbour joining algorithm^55^ in PHYLIP^56^ and the Kimura two-parameter model in MEGA6^57^. Evolutionary distances calculated in neighbour joining analysis were based on a model described by Jukes *et al*.^58^. Tree topologies based on the neighbour joining analysis were evaluated by bootstrap resampling with 1,000 replicates, using the SEQBOOT and CONSENSE programs in the PHYLIP suite.

### Ethics approval

Participation was voluntary, and written informed consent was obtained from all participants. The Institutional Review Board of the Chung-Ang University College of Medicine approved the protocol of this study (IRB number #2010-10-02).

## Electronic supplementary material

Supplementary Fig. S1
