# Supplementary material for: Emergence of Human G2P[4] Rotaviruses in the Post-vaccination Era in South Korea: Footprints of Multiple Interspecies Re-assortment Events
Source: Sci Rep. 2018 Apr 16;8:6011. doi: 10.1038/s41598-018-24511-y (PMC5902508; doi:10.1038/s41598-018-24511-y)
Supplement: Supplementary file 1 — Supplementary Fig. S1 [file 41598_2018_24511_MOESM1_ESM.doc]

**Supplementary information for manuscript:**

**Emergence of Human G2P[4] Rotaviruses in the Post-vaccination Era in South Korea: Footprints of Multiple Interspecies Re-assortment Events**

Hien Dang Thanh,^1^ Van Trung Tran,^1^ Inseok Lim,^2^ & Wonyong Kim^1^*

^1^Department of Microbiology, Chung-Ang University College of Medicine, Seoul 06974, South Korea

^2^Department of Pediatrics, Chung-Ang University College of Medicine, Seoul 06974, South Korea


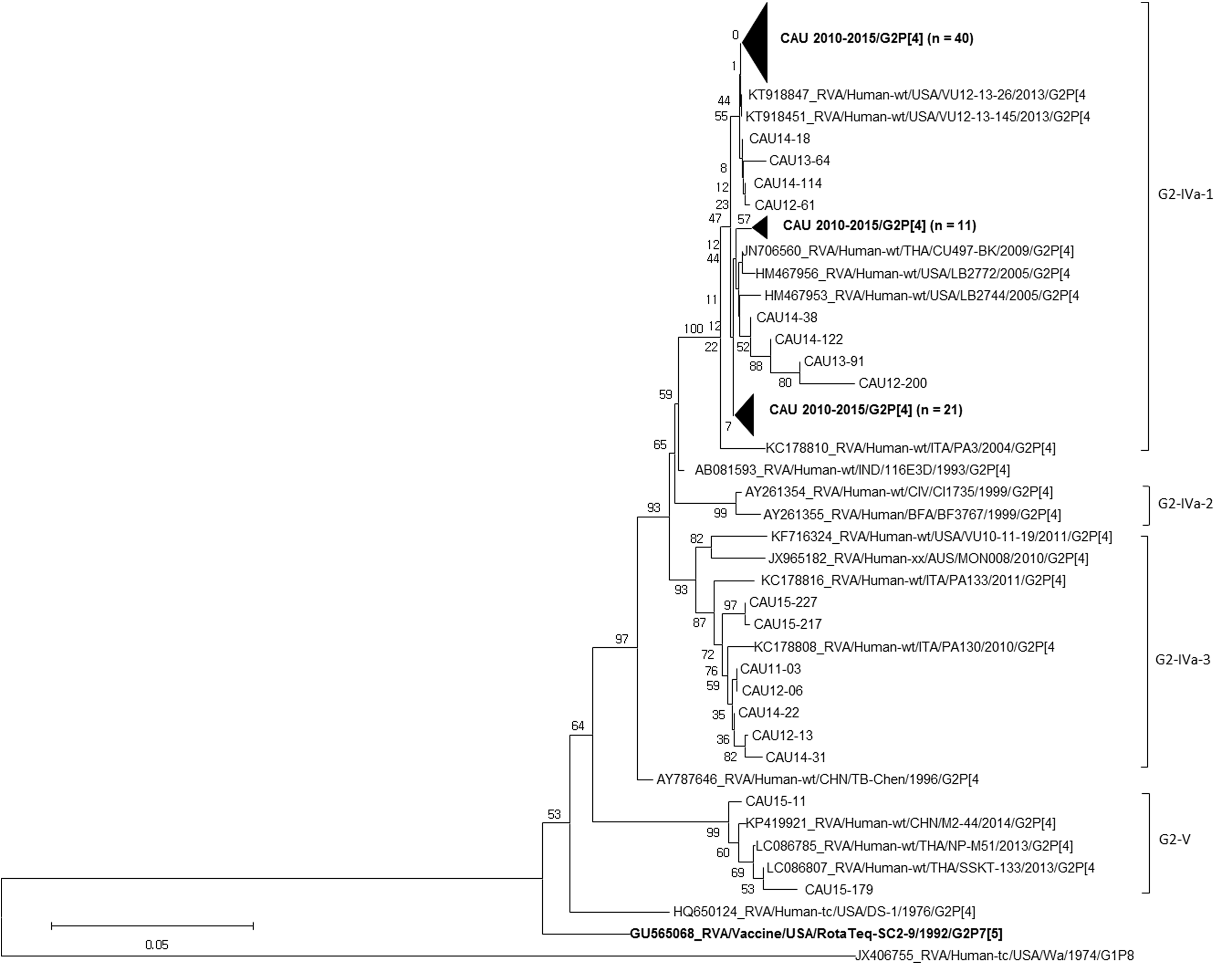


**Figure S1. Phylogenetic tree based on partial VP7 sequences (≥ 500 nt in length) of G2P[4] strains circulating in Seoul, South Korea, from 2010 to 2015. Strain G1P[8] Wa was used as an outgroup.**
